# Current ICD10 codes are insufficient to clearly distinguish acute myocardial infarction type: a descriptive study

**DOI:** 10.1186/1472-6963-13-468

**Published:** 2013-11-07

**Authors:** Roxana Alexandrescu, Alex Bottle, Brian Jarman, Paul Aylin

**Affiliations:** 1 Dr. Foster Unit at Imperial College, Department of Primary Care and Public Health, School of Public Health, Imperial College London, London EC1A 9LA, UK

**Keywords:** ST elevation myocardial infarction, Non-ST elevation myocardial infarction, ICD10

## Abstract

**Background:**

Acute myocardial infarction (AMI) type is an important distinction to be made in both clinical and health care research context, as it determines the treatment of the patient as well as affecting outcomes. The aim of the paper was to determine the feasibility of distinguishing AMI type, either ST elevation myocardial infarction (STEMI) or non-ST elevation myocardial infarction (NSTEMI), using ICD10 codes.

**Methods:**

We carried out a retrospective descriptive analysis of hospital administrative data on AMI emergency patients in England, for financial years 2000/1 to 2009/10. We used the performance of an angioplasty procedure on the same day and on the same or next day of hospital admission as a proxy for STEMI.

**Results:**

Among the ICD10 AMI subcategories, there were inconsistent trends, with some of the codes exhibiting a gradual decline (such as I21.0 Acute transmural myocardial infarction of anterior wall, I21.1 Acute transmural myocardial infarction of inferior wall, I22.0 Subsequent myocardial infarction of anterior wall and I22.1 Subsequent myocardial infarction of inferior wall) and other codes an increase (in particular I21.9 Acute myocardial infarction, unspecified and I22.9 Subsequent myocardial infarction of unspecified site). With the exception of the codes I21.4 Acute subendocardial myocardial infarction, I21.9 Acute myocardial infarction, unspecified, I22.8 Subsequent myocardial infarction of other sites and I22.9 Subsequent myocardial infarction of unspecified site, all the other AMI subcategories appear to have undergone a significant increase in the number of angioplasty procedures performed the same or the next day of hospital admission from around 2005/6. There appear to be difficulties in accurately identifying the proportion of STEMI/NSTEMI by sole reliance on ICD10 codes.

**Conclusions:**

We suggest as the best sets of codes to select STEMI cases I21.0 to I21.3, I22.0, I22.1 and I22.8; however, without any further adaptations, ICD10 codes are insufficient to clearly distinguish acute myocardial infarction type.

## Background

It is widely accepted that it is important to distinguish between acute myocardial infarction (AMI) type as it determines the clinical treatment of the patient [1,2] and affects outcomes [3]. The universal definition of myocardial infarction states that the term should be used ‘whenever there is evidence of myocardial necrosis in a clinical setting consistent with myocardial ischemia’ [1]. In this context, the patients presenting with ischaemic symptoms and persistent electrocardiographic (ECG) ST-segment elevation are categorised as ST elevation myocardial infarction (STEMI). Non-ST segment elevation myocardial infarction (NSTEMI) is based upon the diagnosis of infarction without the ST-segment elevation at the time of symptoms. Of note, whereas STEMI diagnosis relies on ECG, the diagnosis of NSTEMI is more complex, and ultimately depends upon elevation of cardiac markers documenting myocardial injury. It is estimated the great majority of cases are NSTEMI, the ratio of NSTEMI to STEMI being at least 2:1 [4].

Management of STEMI patients includes angioplasty, clot-busting medication and coronary artery bypass graft surgery, with angioplasty performed under strict 90 minutes call-to-balloon time requirements being the first choice for the treatment of these patients. The standard treatment for NSTEMI patients is medical therapy with antiplatelet and anticoagulant medications. However, “early invasive” strategy, i.e., coronary angioplasty performed within the first 96 hours of first admission to hospital, can be part of the treatment for NSTEMI patients who have an intermediate or higher risk of adverse cardiovascular events [2]. The most recent data in England 2011/2012, suggest an increase over time with rates up to 62% of STEMI patients receiving primary angioplasty within 120 minutes from calling; only around 20% of NSTEMI patients are referred for angiography (with follow-on angioplasty if indicated) within 24 hours of admission [4].

Usually the AMI type, either STEMI or NSTEMI, is assessed from clinical information from the medical records. However, most research studies rely on administrative databases that use International Classification of Diseases (ICD) to select and categorise diagnoses. With the exception of the United States, Portugal, Spain and Italy, the only countries with modern health care systems using ICD Ninth Revision, (i.e., ICD9-Clinical Modification (ICD9-CM)), all the other countries use ICD Tenth Revision (ICD10) for coding purposes [5,6]. Whereas the ICD9-CM has separate codes for STEMI and NSTEMI [7], the standard ICD10 classification lacks these specific codes. Of note, a more detailed classification has been released in 2012 (ICD-10 - Clinical Modification (ICD-10-CM)) which explicitly discriminates STEMI/NSTEMI [8]. However, ICD-10-CM is not currently in use in all countries and retrospective studies that include data prior to 2013 will still need to differentiate STEMI and NSTEMI subtypes using the original ICD-10 coding framework. Research in the UK has made use of a classification that identifies STEMI-related hospitalisations as patient records with an ICD-10 primary diagnosis code of I21.0, I21.1, I21.2, I22.0, I22.1 or I22.8 with ‘no record of a previous STEMI hospitalisation within the 28 days prior to admission’ [9,10]. Other recent research, undertaken within an international context, makes use of a slightly different classification for STEMI using ICD10 codes (i.e., I21.0 to I21.3) [11]. These are informal, arbitrary classifications without rigorous validation. The aim of the current analysis was to determine the feasibility of distinguishing AMI type using an empirical approach of categorising 4-digit ICD10 codes.

## Methods

Data were from the Hospital Episodes Statistics (HES) for the financial years from 2000/1 to 2009/10. Hospital Episodes Statistics are administrative data containing information on all admissions to English National Health Service hospitals [12]. Each record in the database represents a finished consultant episode, i.e., the continuous period during which an inpatient is under care of the same consultant. The datasets contain patient and clinical information, the primary and secondary diagnosis fields being coded using ICD10. To avoid multiple counting, we linked episodes of care into admissions (spells) and admissions were linked together if the patient was transferred to another hospital (super-spells). Acute myocardial infarction was defined as admission to hospital with AMI as primary diagnosis. The ICD-10 codes used to define AMI were I21-I22, assigned for each spell based on the primary diagnosis in the first episode of care or, if the primary diagnosis was a vague symptom or sign, we used the second episode to derive the diagnosis. All emergency admissions (2000/1 to 2009/10) in England for AMI patients have been extracted based on these criteria.

To determine the feasibility of distinguishing AMI type (STEMI vs. NSTEMI) using ICD10 subcategories codes, we present coronary angioplasty procedure use stratified by AMI ICD10 subcategories, over the study period (2000/1 to 2009/10). Data for angioplasty procedures were selected based on codes K49, K50 and K75 from the Office of Population Censuses and Surveys Classification of Surgical Operations and Procedures, fourth revision (OPCS 4), occurring in any procedure field in any episode. Furthermore, based on the number of days between the date of angioplasty and the date of hospital admission, we selected two groups of AMI patients: those undergoing coronary angioplasty on the ‘same day’ and on the ‘same or next day’ of hospital admission. These are procedures that are performed in clinical emergency scenarios. The ‘same or next day’ data has been used as a sensitivity analysis for the ‘same day’ data on angioplasty procedures. We hypothesised that the great majority of these procedures will be found predominantly in STEMI patients.

The percentages of AMI cases undergoing coronary angioplasty have been computed by dividing the number of procedures performed during a given year by the total number of AMI patients (overall or within each specific subcategory code) for that year. Table 1 presents a description of the ICD10 AMI codes and the corresponding OPCS 4 coronary angioplasty codes.

**Table 1:** ICD10 diagnosis codes for AMI and the OPCS 4 codes for coronary angioplasty

**Code**	**Description**
ICD10	
I21	Acute myocardial infarction
I21.0	Acute transmural myocardial infarction of anterior wall
I21.1	Acute transmural myocardial infarction of inferior wall
I21.2	Acute transmural myocardial infarction of other sites
I21.3	Acute transmural myocardial infarction of unspecified site
I21.4	Acute subendocardial myocardial infarction
I21.9	Acute myocardial infarction, unspecified
I22	Subsequent myocardial infarction
I22.0	Subsequent myocardial infarction of anterior wall
I22.1	Subsequent myocardial infarction of inferior wall
I22.8	Subsequent myocardial infarction of other sites
I22.9	Subsequent myocardial infarction of unspecified site
OPCS 4	
K49	Transluminal balloon angioplasty of coronary artery
K50	Other therapeutic transluminal operations on coronary artery
K75	Percutaneous transluminal balloon angioplasty and insertion of stent into coronary artery

OPCS 4 Office of Population Censuses and Surveys Classification of Surgical Operations and Procedures, fourth revision.

In addition, a comparison was performed between our study population and the Myocardial Infarction National Audit Project (MINAP) database, the largest clinical observational dataset of patients from England and Wales hospitalised with an acute coronary syndrome [13]. MINAP data cover all acute hospitals that admit coronary syndrome patients and, for each patient, includes detailed clinical information (e.g., investigation, results and treatment). Of note, some of the hospitals do not report all their data due to lack of resources. As a result, in practice, MINAP dataset comprises the great majority of STEMI patients and only a part of NSTEMI patients. The data availability has restricted the comparison of STEMI and NSTEMI cases to aggregate figures covering England and Wales and the time period 2003 to 2009. For the purpose of this analysis, HES data, reported on standard financial year basis, were allocated to calendar years. Data manipulation and analysis were performed using SAS (v9.1).

Study approval: We have permission from the NIGB under Section 251 of the NHS Act 2006 (formerly Section 60 approval from the Patient Information Advisory Group) to hold confidential data and analyse them for research purposes. We have approval to use the data for research and measuring quality of delivery of healthcare, from the South East Ethics Research Committee.

## Results

The study population consisted of 716317 AMI admissions over 2000/1 to 2009/10, 599485 Acute myocardial infarction I21 (83.7%) and 116832 Subsequent myocardial infarction I22 (16.3%). The commonest I21 subcode was I21.9 (site unspecified, 43.3%) whereas within I22 the corresponding code was I22.9 (61.4%).

**Figure 1:**
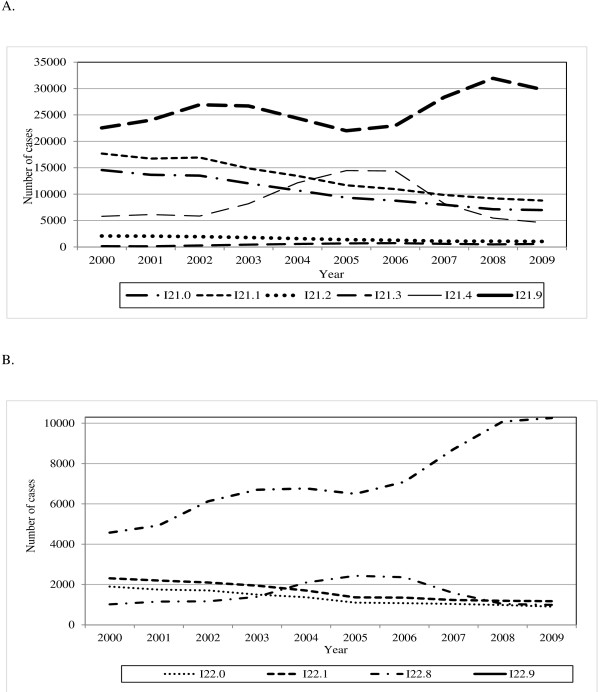
**Trends in the number of inpatients stratified by AMI ICD10 codes, HES data, England 2000/1-2009/10. (A)** Acute Myocardial Infarction I21. **(B)** Subsequent Myocardial Infarction I22.

Figure 1A and 1B present trends over 2000/1 to 2009/10 in the number of AMI cases stratified by subcategory codes. Within I21, the number of cases coded within subcategories I21.0 to I21.2 gradually declined from 34320 in 2000/1 to 16793 in 2009/10. Much larger fluctuations were exhibited by I21.4 and I21.9 cases, with an overall tendency to increase. Within subsequent myocardial infarction, I22, the number of I22.0 and I22.1 cases gradually declined over time. By contrast, the number of I22.8 and, in particular, I22.9 cases increased over the same study period.

**Figure 2:**
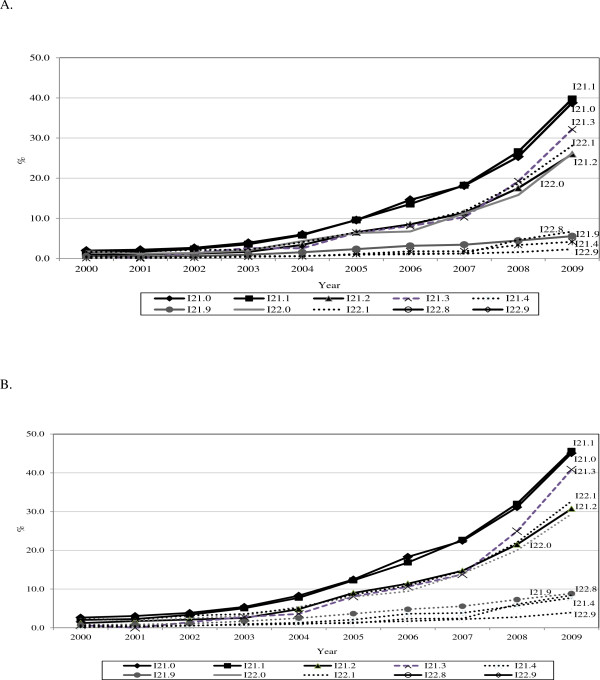
**Use of angioplasty (% out of total cases) stratified by AMI ICD10 codes, HES data, England 2000/1-2009/10. (A)** Angioplasty performed same day of hospital admission. **(B)** Angioplasty performed same or next day of hospital admission.

Over the study period there was an increase in the percentage of AMI cases undergoing coronary angioplasty on the same day and on the same or next day of hospital admission. In 2009/10, 14.4% and 18.2% of AMI cases underwent coronary angioplasty on the same day and on the same or next day (data not shown). Analysis stratified by subcategories codes (Figure 2A and 2B) shows that, apart from the codes I21.4, I21.9, I22.8 and I22.9, all the other AMI subcategories appear to have undergone a significant increase in the number of angioplasty procedures performed on the same day or on the same or next day of hospital admission, in particular starting with the second part of the study, up to values of minimum 26% for same day angioplasty and 29% for same or next day angioplasty (i.e., both I21.2 and I22.0). Of note, the increase in AMI cases subcategory I22.8 was restricted to the last two-year study period.

**Figure 3:**
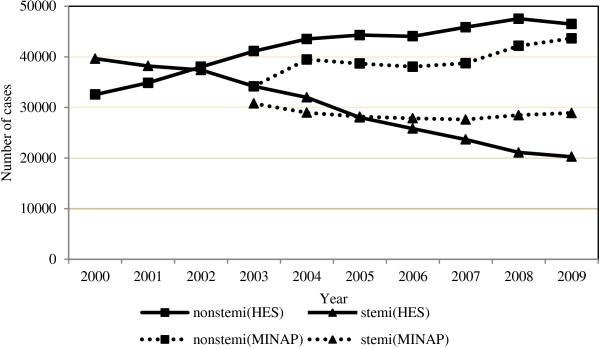
Comparison of HES data with MINAP data stratified by AMI type, 2000-2009.

A comparison of HES data with MINAP database (Figure 3) shows similar patterns in the temporal changes in the number of cases for NSTEMI, although the two sets of figures differ by an almost constant amount of cases. The temporal pattern of STEMI cases is different between HES (decreasing) and MINAP data that is rather stable. Of note, MINAP data cover both England and Wales whereas our study population is restricted to England alone.

## Discussion

We undertook a descriptive analysis of the trends in diagnosis codes and angioplasty procedure use of the AMI cases stratified by ICD10 subcategory codes to determine the feasibility of the ICD10 codes to distinguish between STEMI and NSTEMI.

Among the ICD10 AMI subcategories, the trends were complex. The analysis clearly shows a gradual decline of AMI cases coded as I21.0 and I21.1 as well as I22.0 and I22.1 and an increase in AMI cases coded I22.8 and I22.9. Research undertaken in England (using the classification described by Pereira et al) as well as elsewhere (data relying on ICD-9CM) [10,13-15] has shown a reduction of STEMI cases and an increase in NSTEMI cases. Possible explanations for the increase in NSTEMI cases include use of the revised definition for an acute MI and the widespread use of troponin as a (more sensitive) marker for diagnosing AMI since 2005 (i.e., when its use became universal in England and Wales) [10,14-16]. The previous UK classification identified STEMI-related hospitalisations as patient records with an ICD-10 primary diagnosis code of I21.0, I21.1, I21.2, I22.0, I22.1 or I22.8. Based on our analysis, these are indeed the codes showing some decrease in the number of cases, whereas the remaining ones, possible NSTEMI cases, showed an (expected) increase in data reporting. Although the findings reflect what we would expect with regard to STEMI/NSTEMI cases, from this part of the analysis it is difficult to draw the conclusion that certain ICD-10 diagnosis codes I21.0, I21.1, I21.2, I22.0, I22.1 or I22.8 are STEMI cases.

We have used data on coronary angioplasty performed same day as well as same or next day of hospital admission to capture a great majority of procedures that are performed in clinical emergency scenarios. Assuming accurate classification by AMI type, we hypothesised that coronary angioplasty procedures performed within the same day of hospital admission will be found predominantly in STEMI patients. The results show several AMI subcategories appear to have undergone a significant increase in the number of angioplasty procedures same day/same or next day of admission, either starting with the second part of the study period (I21.0 to I21.3, I22.0 and I22.1) or only over the last two study years (I22.8). Apart from I21.3, all these previously mentioned ICD10 codes have been previously categorised as STEMI patients, a classification that seems to be in agreement with the results of the present analysis. However, the results also clearly suggest I21.3 falling into the STEMI category. Of note, I21.3 has been included in the category STEMI within some other international research [11]. The MINAP 2010 report gives approximately 45% of STEMI patients receiving primary angioplasty in England and Wales over 2009/2010 [10]. The corresponding figures in our study (using I21.0 to I21.2, I22.0, I22.1, and I22.8 data over 2009) were 35.7% (same day angioplasty) and 41.3% (same or next day angioplasty). Interestingly, I21.9, representing the great majority of I21 cases, exhibited only moderate values of angioplasty use (under 10%). A further analysis of the socio-demographic characteristics of the non-specific codes I21.9 and I22.9 suggests that, compared with the rest of the AMI cases, these patients tend to be older (age mean (SD), years 70.1 (13.5) and 73.1 (11.5) vs. 66.1 (13.6), p < 0.0001) and have more comorbidity conditions (Charlson index score mean (SD), 0.96 (1.0) and 1.5 (0.9) vs. 0.81 (0.9), p < 0.0001), data not shown. The literature shows that NSTEMI vs STEMI patients tend to be older and have higher comorbidity scores [3], a finding that further supports our assumption related to the I21.9 and I22.9 codes. However, we have also found a fewer proportion of I21.9 and I22.9 patients have been treated in a cardiology ward compared with the rest of the AMI patients that might suggest poor diagnosis or can explain the lower rates of angioplasty performed within these two categories of patients (cardiology ward of treatment 15.3% and 15.7% vs. 25.8%, p < 0.000), data not shown.

The analysis leads to the conclusion that it is indeed difficult to distinguish AMI type using the terminology included in standard ICD10 without any other further adaptations. However, we suggest as the best sets of codes to select STEMI cases I21.0 to I21.3, I22.0, I22.1 and I22.8. Table 2 presents the ICD9 CM and ICD10 CM including the new versions that differentiate STEMI/NSTEMI and the ICD10 classification with our proposed description. It is noteworthy, within the new version of ICD10 that comes into effect in October 2013 (ICD10-CM) STEMI and NSTEMI are mutually exclusive based on the coding subcategories [8]. ICDS-10-CM is the coding system developed in the United States, so the existence of these codes does not mean that in the UK, it will be implemented some time soon. The transition to a new ICD coding system might be seen more of a future, long term solution to the problem of identifying STEMI and NSTEMI, rather than an immediate one.

**Table 2:** Relevant ICD 9 and ICD10 diagnosis codes for AMI

**ICD**							
	**ICD9**		**ICD10**			**ICD10-CM**	
**Code**	**Previous description**	**New description (October 2005)**	**Code**	**Current description**	**Proposed description**	**Code**	**Description**
410.01	Anterolateral wall	STEMI of anterolateral wall	I21	Acute myocardial infarction		I21	ST elevation (STEMI) and non-ST elevation (NSTEMI) myocardial infarction
410.11	Other anterior wall	STEMI of other anterior wall	I21.0	Acute transmural myocardial infarction of anterior wall	STEMI	I21.0	ST elevation (STEMI) myocardial infarction of anterior wall
410.21	Inferolateral wall	STEMI of inferolateral wall	I21.1	Acute transmural myocardial infarction of inferior wall	STEMI	I21.1	ST elevation (STEMI) myocardial infarction of inferior wall
410.31	Inferoposterior wall	STEMI of inferoposterior wall	I21.2	Acute transmural myocardial infarction of other sites	STEMI	I21.2	ST elevation (STEMI) myocardial infarction of other sites
410.41	Other inferior wall	STEMI of other inferior wall	I21.3	Acute transmural myocardial infarction of unspecified site	STEMI	I21.3	ST elevation (STEMI) myocardial infarction of unspecified site
410.51	Other lateral wall	STEMI of other lateral wall	I21.4	Acute subendocardial myocardial infarction	NSTEMI	I21.4	Non-ST elevation (NSTEMI) myocardial infarction
410.61	True posterior wall	STEMI of true posterior wall	I21.9	Acute myocardial infarction, unspecified	NSTEMI	---	-----------------------------------------------------
410.71	Subendocardial	NSTEMI	I22	Subsequent myocardial infarction		I22	Subsequent ST elevation (STEMI) and non-ST elevation (NSTEMI) myocardial infarction
410.81	Other specified sites	STEMI of other specified sites	I22.0	Subsequent myocardial infarction of anterior wall	STEMI	I22.0	Subsequent ST elevation (STEMI) myocardial infarction of anterior wall
410.91	Unspecified site	Myocardial infarction NOS	I22.1	Subsequent myocardial infarction of inferior wall	STEMI	I22.1	Subsequent ST elevation (STEMI) myocardial infarction of inferior wall
			---	-------------------------------	---------	I22.2	Subsequent non-ST elevation (NSTEMI) myocardial infarction
			I22.8	Subsequent myocardial infarction of other sites	STEMI	I22.8	Subsequent ST elevation (STEMI) myocardial infarction of other sites
			I22.9	Subsequent myocardial infarction of unspecified site	NSTEMI	I22.9	Subsequent ST elevation (STEMI) myocardial infarction of unspecified site

STEMI ST elevation myocardial infarction; NSTEMI non- ST elevation myocardial infarction; NOS not otherwise specified.

We acknowledge that is difficult to compare HES data with MINAP records, except in aggregate [13]. Interestingly, the number of STEMI cases seems to be lower compared with MINAP STEMI data for most of the study period. One possible explanation might relate to the data coverage. Nevertheless, since Wales’s data might account for up to 10% of AMI records, it is unlikely that this would explain the entire differences in our comparison [17]. Perhaps an appropriate segregation of ‘real’ STEMI from NSTEMI cases within the large AMI subcategory I21.9 (currently coded as NSTEMI) would make some contribution in reducing the differences between MINAP and our study population. However, in the absence of the clinical data this is less likely to be achievable. With regard to the number of NSTEMI cases, this seems to be higher compared with MINAP NSTEMI data. This is unsurprising, considering that MINAP will not record all patients having NSTEMI.

We used hospital administrative data that is limited by the potential of misclassification bias in assigning patients to AMI subcategories as well as in relation o how diagnoses are recorded by different hospitals. Moreover, the lack of a gold standard with individual level data prevented a true validation of the ICD-10 codes to differentiate STEMI from NSTEMI. In this context it worth noting research shows that even when specific codes to differentiate STEMI from NSTEMI exists, i.e., ICD9-CM, the correlation between ECG diagnoses and ICD-9 codes is high, but there is still room for improvement [18]. The disagreement has been attributed to a combination of factors including the training of coding staff, the information available on medical records, the ambiguity of some AMI cases or even the accuracy of the coding classification itself, suggesting the need for collective efforts from a wide range of health care professionals in providing accurate information.

Within countries that use ICD10 (or country-specific versions of this classification), different approaches have been implemented to distinguish between STEMI and NSTEMI. In Scotland, coding guidelines have been recently updated by adding a 5^th^ digit for use only with AMI codes, while in Canada, as an interim solution, ICD10-CA has expanded a separate subcategory to capture relevant information in this regard [19,20]. Nevertheless, in Wales for example, no changes to the ICD10 codes have been made and, as a consequence, AMI type is assessed based on further clarification from the clinician [21].

## Conclusions

Our research suggests that there are difficulties in identifying accurate data on the proportion of STEMI/NSTEMI by sole reliance on ICD10 codes. However, we suggest the best sets of codes to select STEMI cases are I21.0 to I21.3, I22.0, I22.1 or I22.8.

## Consent

Written informed consent was obtained from the patient for the publication of this report and any accompanying images.

## Competing interests

The authors declare that they have no competing interests.

## Authors’ contributions

RA and PA participated in the conception and design of the study. RA carried out the analysis. All authors participated in interpretation of data. RA and PA have been involved in drafting the manuscript; AB and BJ have been involved in revising the manuscript critically for important intellectual content. All authors read and approved the final manuscript.

## Pre-publication history

The pre-publication history for this paper can be accessed here:

http://www.biomedcentral.com/1472-6963/13/468/prepub
